# The PREP pipeline: standardized preprocessing for large-scale EEG analysis

**DOI:** 10.3389/fninf.2015.00016

**Published:** 2015-06-18

**Authors:** Nima Bigdely-Shamlo, Tim Mullen, Christian Kothe, Kyung-Min Su, Kay A. Robbins

**Affiliations:** ^1^Syntrogi Inc. San Diego, CA, USA; ^2^Swartz Center for Computational Neuroscience, University of California San Diego, La Jolla, CA, USA; ^3^Department of Computer Science, University of Texas at San Antonio San Antonio, TX, USA

**Keywords:** EEG, artifact, preprocessing, EEGLAB, BCILAB, machine learning, big data

## Abstract

The technology to collect brain imaging and physiological measures has become portable and ubiquitous, opening the possibility of large-scale analysis of real-world human imaging. By its nature, such data is large and complex, making automated processing essential. This paper shows how lack of attention to the very early stages of an EEG preprocessing pipeline can reduce the signal-to-noise ratio and introduce unwanted artifacts into the data, particularly for computations done in single precision. We demonstrate that ordinary average referencing improves the signal-to-noise ratio, but that noisy channels can contaminate the results. We also show that identification of noisy channels depends on the reference and examine the complex interaction of filtering, noisy channel identification, and referencing. We introduce a multi-stage robust referencing scheme to deal with the noisy channel-reference interaction. We propose a standardized early-stage EEG processing pipeline (PREP) and discuss the application of the pipeline to more than 600 EEG datasets. The pipeline includes an automatically generated report for each dataset processed. Users can download the PREP pipeline as a freely available MATLAB library from https://github.com/VisLab/EEG-Clean-Tools.

## Introduction

Traditional EEG experiments typically collect data from a limited number of subjects in a controlled environment with the purpose of discovering similarities and differences in subject response as a few experimental parameters are varied. Recent improvements in technology and recognition of the potential applications of real-world brain imaging using EEG supported by physiological monitoring are shifting experimental paradigms toward large-scale data collection under more loosely controlled conditions (Liao et al., 2012; Ortiz-Rosario and Adeli, 2013).

In addition to technology advances, the recognition of the significant inter- and intra-subject variability in EEG, as well as the variety of possible environmental interactions that might significantly affect brain behavior, have given impetus to large-scale testing of generalizability across subjects and paradigms. On the analysis side, new methods in machine learning rely on the transfer of knowledge from data acquired under one set of conditions to learn patterns or to classify data acquired in another situation. Large-scale use of transfer learning requires “interoperability” of data collections. The machine learning community has also recognized that most algorithms require large amounts of data in order to achieve the prediction accuracies needed for real-world applications.

Efforts to pool data acquired under a variety of experimental conditions face several roadblocks due to lack of standards for data preparation. Researchers must worry about the accuracy of the time synchronization, particularly for experiments recording additional data streams in conjunction with EEG. Real-world imaging, and longer recording sessions in general, are prone to many more “technical errors” as well as to subject-generated artifacts. Finally, the original experimenter is likely to have retained multiple versions of the data preprocessed in an undocumented manner.

The importance of standardization and automatization is starting to be recognized (Keil et al., 2014). Gross et al. (2013) lay out extensive guidelines for handling MEG data, including recommendations for processing approaches, caveats, and reporting requirements for a variety of applications. Although some of these standards are also applicable to EEG, artifact removal and validation of processing approaches remain a long-standing open problem for EEG. While most researchers perform filtering, referencing, and artifact removal prior to further analysis, the process is by no means standardized or automated.

The lack of standardization of data preparation presents a dilemma for researchers who wish to share data or to combine data from multiple collections for analysis. Machine-learning algorithms are notoriously sensitive to preprocessing and feature normalization. Common transformation techniques such as independent component analysis (ICA) are also quite sensitive to data preparation. Although features such as correlation or spectral features can be somewhat self-normalizing, amplitude-based features often vary dramatically in scale from headset to headset and from session to session. While normalization can reduce cross session/subject/headset variations, normalization of data contaminated by large experimentally generated artifacts does not generally improve the signal-to-noise ratio and frequently results in mis-training and poor performance from machine learning algorithms.

This paper reports on our efforts to develop a standardized, completely automated and hopefully non-controversial early-stage preprocessing pipeline (the PREP pipeline) that detects and removes certain experimentally generated artifacts. We distinguish externally generated experimental artifacts such as electrical interference from subject-generated artifacts such as eye blinks or muscle activations, which may contain information about subject state. Researchers could remove these subject-generated artifacts by applying additional processing pipelines to the preprocessed data.

A primary goal of this work is to produce EEG data in a standardized, “depositable” format suitable for large-scale downstream analysis across multiple collections. By “depositable” we mean a format that analysts would find acceptable as input for most applications in lieu of raw data. Many of the decisions made are open to debate. However, adoption of some standardized format is necessary for progress in large-scale machine learning applications of EEG. Raw data provides too rough and undocumented an interface for effective automated downstream processing. Two key decisions support this effort: the pipeline should be completely automated and the repository should maintain the original data in a format suitable for input into this pipeline. Thus, if the pipeline is later modified, the owners of the repository can rerun the processing and produce a new fully documented release of the repository.

The depositable preprocessing pipeline consists of three steps: perform an initial clean-up, determine and remove a robust reference signal, and interpolate the bad channels (channels with a low recording SNR). The output consists of the EEG data saved in an EEGLAB EEG structure along with auxiliary files to make the events, channels and metadata easily available for input in systems other than MATLAB. We provide source functions, built on standard HDF5 libraries to read both the data and the metadata in MATLAB, R, Python, Java, and C. In this way, users can import data into other systems for computing. Users can run the preprocessing through EEGLAB as a plugin, as a standalone function, or as part of the containerized pipeline.

The PREP pipeline has an additional reporting capability that produces a pdf document for each data set. The report summarizes the dataset characteristics and identifies potentially bad sections of the signal for further analysis. The pipeline uses some simple heuristics that allow researchers to assess quickly whether a particular dataset might have issues. When dealing with hundreds or potentially thousands of datasets, such a capability is important. When a particular dataset shows anomalous downstream results, the researcher can examine the reports for unusual features or behavior that would indicate experimental artifacts. Additional utility functions provide summaries of an entire data collection and identify potential issues. The following subsections discuss each step (initial clean-up, referencing, and interpolation) in more detail. We found a complicated interaction between high-pass filtering, line noise removal, and referencing, which we describe below in more detail.

The remainder of this paper describes the proposed PREP preprocessing pipeline, explains the reasoning behind the decisions made at each point, and demonstrates the effectiveness of the approach in a variety of situations. Section Early Stage Preprocessing (PREP) describes the pipeline components. Section Reporting and Some Example Results shows examples of the types of anomalies that can occur in individual datasets, and Section Summary Measures presents examples of collection summaries. Section Other Tests reports the results of applying the pipeline to EEG with synthetically generated bad channels and examines the downstream effects of the pipeline on classification. Section Discussion summarizes the results and offers some concluding remarks. It should be emphasized that this pipeline only performs very early-stage preprocessing and does not preclude additional automated preprocessing. We return to this issue in Section Discussion.

## Early stage preprocessing (PREP)

The philosophy of the PREP pipeline is to perform preprocessing needed to standardize the data into a form that is useful for a variety of applications, while preserving as much of the signal as possible. Unfortunately, specialization and standardization may conflict with respect to collection development, and there can be a complex interaction between exact choice of preprocessing such as filtering and downstream behavior (Widmann and Schröger, 2012). On the other hand, providing collections that only have raw data and no standardized preprocessed data is problematic for exactly the same reason.

A major goal of large-scale collection development is to test the robustness of approaches and to compare neurological phenomena across subjects and experiments. Such comparisons need to start from well-documented analysis-ready base data sets. A critical step for automated large-scale processing is the identification and removal of bad channels, since many algorithms will fail in the face of egregiously bad signals. As described below, there is a complicated interaction between bad channels and referencing. PREP performs automated noise removal, bad channel detection, and referencing in a way that allows users to specialize the data to particular applications without having to work with the raw data.

A summary of the PREP pipeline is:

1. Remove line-noise without committing to a filtering strategy.
2. Robustly reference the signal relative to an estimate of the “true” average reference.
3. Detect and interpolate bad channels relative to this reference.
4. Retain sufficient information to allow users to re-reference using another method or to undo interpolation of a particular channel.

While the steps seem simple, we demonstrate the variety of issues that arise when these steps are not performed uniformly across datasets. The PREP pipeline also provides very detailed summary information and visualizations that allow researchers to identify unusual features at a glance. The remainder of this section discusses the components of the PREP pipeline and their interactions.

### Line noise removal

Many analysts automatically perform a notch filter at 60 Hz to remove line noise. Such notch filters often use a notch width of 10 Hz or larger, resulting in significant signal distortion in frequencies between 50 and 70 Hz. This distortion should not be an issue for analyses that immediately apply a low-pass filter to the signal, say at 40 Hz, but may preclude certain high-frequency studies. Some studies have also shown that non-causal low-pass filtering can significantly change ERP onsets (Vanrullen, 2011; Rousselet, 2012). In addition, Barnett and Seth (2011) have shown that filtering, particularly low pass filtering, can have a damaging impact on Granger causality and other connectivity computations.

Mitra and Pesaran (1999) suggest a multi-taper decomposition approach for identifying and removing line noise components while minimizing background signal distortion. We leverage routines from the *cleanline* EEGLAB plugin developed by Mullen (2012), which extends functionality from the open source Chronux toolbox (Mitra and Bokil, 2007). This method traverses the data using a short sliding window (4 s with a 1-s slide by default). The method transforms the data in each window into the frequency domain using a set of Slepian or multi-tapers with a predetermined spectral resolution. Such tapers are ideal for isolating spectral energy within frequency bands, even for short time windows. By default, we use a taper bandwidth (*TBW*) of 2 Hz in each 4-s sliding window (*W*), which contains *N* sample points. The Slepian tapers are created by calling dpss(N, TBW^*^W/2, TBW^*^W–1) where *dpss* is the discrete prolate spherical sequence function from the MATLAB Signal Processing Toolbox.

The *cleanline* method fits a frequency-domain regression model to estimate the amplitude and phase of a deterministic sinusoid of a specified frequency, embedded in locally white noise. This is an idealized model of sinusoidal line noise of unknown phase and amplitude. A Thompson *F*-test assesses whether the complex amplitude is significantly non-zero. If the amplitude is significant (*p* < 0.01), the method reconstructs the time-domain sinusoid for the line noise frequencies. The method stitches together results from successive overlapping windows by using a sigmoidal weighted average specified by a smoothing parameter *tau* (100 by default). Finally, the method subtracts this fitted signal from the data. We repeat this process (a maximum of 10 iterations by default) until the sinusoid amplitude for the specified frequencies is not significantly different from the background.

In practice, the exact line frequency is unknown and variable. Following Mitra and Pesaran, we apply the regression model across a range of frequencies centered on each candidate line-noise frequency and select the frequency that maximizes the Thompson F-statistic. The range is +/− *fScanBandwidth*, which is 2 Hz by default. The user must provide a rough estimation of the line frequencies or use the defaults of 60 Hz with harmonics that are multiples of 60 up to the Nyquist frequency. The advantage of this approach over notch filtering is that it removes only deterministic line components, while preserving “background” spectral energy. The sliding window estimation further allows for non-stationarities in the phase and amplitude of the line component. The PREP pipeline function encapsulates this denoising functionality in the function *cleanLineNoise*.

**Figure 1:**
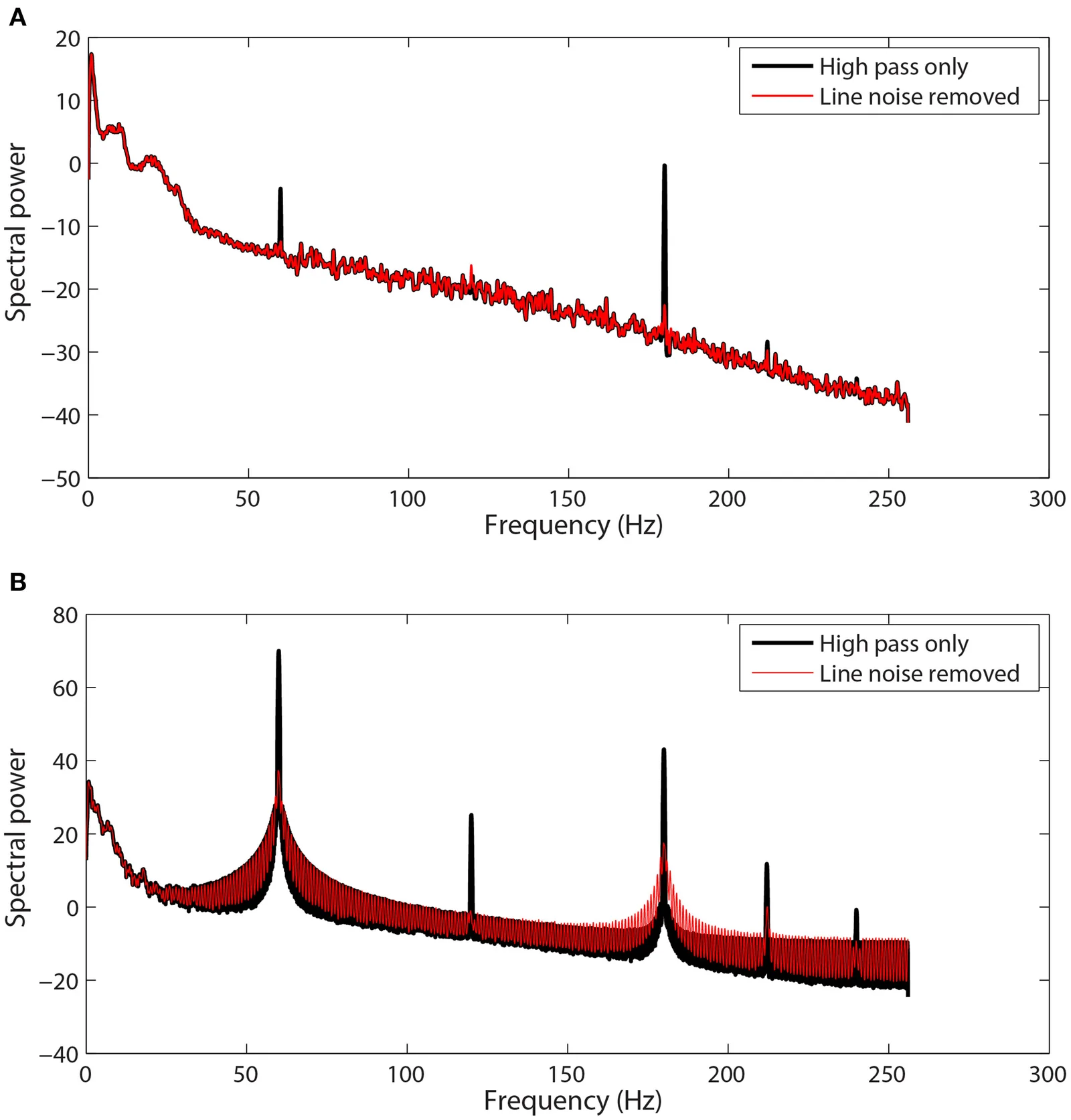
**Selected channel spectra from 64-channel Biosemi EEG before and after line noise removal**. Spectral power is in units of $10{\text{log}}_{10}\left(\mu {\text{V}}^{2}\right)$. Data was high-pass filtered at 1 Hz prior to line noise removal. **(A)** A channel with sharp peaks at 60 and 180 Hz. **(B)** A channel contaminated by non-stationary transformer noise. This channel is interpolated at a later stage in the pipeline.

Figure 1 shows examples of channel spectra before and after application of *cleanLineNoise* for two EEG datasets from the same collection using a Biosemi 64-channel EEG headset. The channel in Figure 1A had narrow noise peaks, and *cleanLineNoise* was able to remove spectral peaks at 60 and 180 Hz below the significance level with little distortion in the overall spectrum.

The *cleanLineNoise* function reduced the 60 and 180 Hz peaks of the channel in Figure 1B, but the algorithm could not deal with the underlying spectral distortions at these frequencies. This channel had narrow spectral peaks at 120 and 240 Hz, which the algorithm did successfully remove. The *cleanLineNoise* function also reduced a designated 212 Hz spectral peak. Although *cleanLineNoise* was not able to clean this spectrum, the corresponding channel failed all of the noisy channel detection criteria at the next stage. Robust referencing subsequently replaced the channel with its interpolated value. The ringing behavior of Figure 1B is typical of channels with large, non-stationary spectral artifacts.

### High-pass filtering-line noise removal interaction

In the original version of the pipeline, we high-pass filtered at 1 Hz before removing line noise. The signals of Figure 1 have been high-pass filtered prior to line-noise removal. However, our users were concerned about the effect of high-pass filtering on ERP and connectivity analyses. These concerns and a series of articles about the perils of filtering for ERP processing (Vanrullen, 2011; Acunzo et al., 2012; Rousselet, 2012; Widmann and Schröger, 2012; Widmann et al., 2014; Tanner et al., 2015) caused us to re-evaluate the approach. We decided that it would be better to perform line noise removal as well as referencing and bad channel interpolation without committing to a particular high-pass filtering strategy. This section discusses the consequences of that strategy.

**Figure 2:**
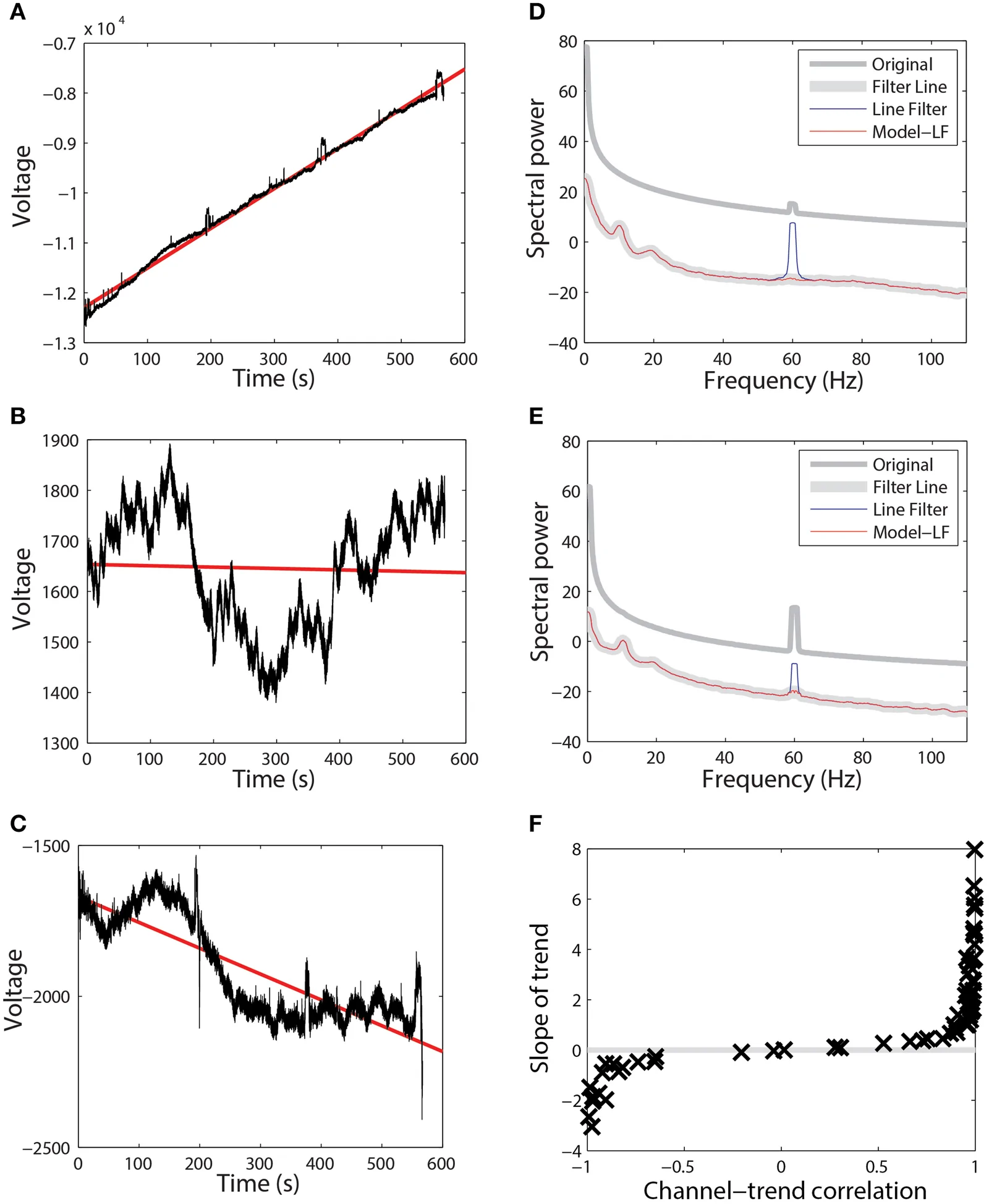
**Raw data from three channels taken from a single session recorded using a 64-channel Biosemi headset**. Voltages are in units of microvolts. Spectral power is calculated in units of $10{\text{log}}_{10}\left(\mu {\text{V}}^{2}\right)$. **(A)** A channel that is highly correlated (0.997) with its linear trend line (shown in red). **(B)** A channel that is weakly anti-correlated (−0.039) with its linear trend line (shown in red). **(C)** A channel that is anti-correlated (−0.838) with its linear trend line (shown in red). **(D)** Power spectrum of the channel shown in **(A)**. Medium gray line corresponds to the spectrum of original data. Thick gray line corresponds to data high-pass filtered at 0.3 Hz followed by line noise removal. Blue line corresponds to data cleaned of line noise then high-pass filtered at 0.3 Hz. Red line corresponds to data where line noise removal and subsequent filtering are applied after removal of the global best-fit linear trend. **(E)** Power spectrum of channel shown in **(B)** shown as in part **(D)**. **(F)** Correlation of all of the channels in this dataset with their best-fit line vs. the slope of that best-fit line.

Extensive tests showed that the line-noise removal algorithms do not perform well if done without prior high-pass filtering or trend removal. As with other Fourier transform approaches, multi-taper spectral estimation assumes signal stationarity, and therefore removal of long-term trend prior to spectral estimation can improve estimation and interpretation of the signal spectrum. EEG signals often exhibit large temporal non-stationarities due to drift, which affect subsequent analyses. Figures 2A–C show three examples (channels 1, 18, and 7) from the same session using a 64-channel Biosemi headset recorded over 10 min. Channel 1, which follows a linear trajectory over the entire period, has a correlation of 0.997 with a best-fit line whose slope is approximately 8. In contrast, channel 18 has a correlation of −0.039 with a best-fit line of slope −0.028 over this period. Channel 18 exhibits large-scale non-stationarities over a shorter time scale than does channel 1. Channel 7, another typical channel, has a correlation of −0.838 and a slope of −0.855.

While the presence of drift in EEG is well known, the lack of consistency of the drift across time and channels and its effect on order of computations and round-off may not be fully-appreciated. Figure 2F shows a plot of the correlation of channel signal with its best-fit line vs. the slope of that line for the 64 EEG channels of this session. The sigmoidal shape is typical across headsets and sessions, however there is no consistency in the details with respect to channel and headset. Some sessions have all channels on one side or the other of the curve, while others are mixed. High-pass filtering removes this non-stationarity, but pipelines that perform any operations prior to high-pass filtering should take this drift into account. In particular, referencing done in single precision prior to high pass filtering is dominated by this effect and does not achieve the desired removal of common noise sources.

These long-term signal non-stationarities also affect the multi-taper line noise removal algorithm. Figures 2D,E show the power spectra corresponding to the signals of Figures 2A,B, respectively. The medium gray line (“Original”) is the power spectrum of the original signal without any processing, while the thick gray line (“Filter Line”) shows the signal after high-pass filtering at 0.3 Hz followed by line noise removal. The blue line shows the result of removing line noise and then performing the high-pass filter. The multi-taper removal algorithm relies on significance testing and cannot detect line noise with sufficient precision when there are large spectral effects due to non-stationarity. When the signal is subsequently high-pass filtered, a large spectral peak remains (“Line Filter”).

The red lines in Figures 2D,E are the result of subtracting the linear trend line from the signal, followed by line noise removal, followed by high-pass filtering (“Model - LF”). After removing the trend, the multi-taper procedure is able to detect and remove line noise adequately.

We obtain essentially the same results if we high-pass the original signal, remove line noise from the high-passed signal, and capture the signal that was removed. We subtract the captured noise signal from the original signal to obtain a “cleaned” unfiltered signal. If we subsequently high-pass filter the cleaned unfiltered signal, we find the line noise has been removed as though the signal had been filtered prior to line noise removal. The result is similar for a 1 Hz high-pass filter. The PREP pipeline uses this strategy for its line noise removal to avoid committing to a filtering strategy for the final pipeline output.

The PREP pipeline uses the EEGLAB *pop_eegfiltnew* function contributed to the EEGLAB distribution by Andreas Widmann for this stage. We use the default filter settings and a 1 Hz high-pass cutoff. We also note that double precision computation is essential because round off in single precision quickly destroys any natural commutativity of the linear operations. Many EEGLAB functions routinely call the EEGLAB *eeg_checkset* function, which converts EEG data to single precision by default. A user can override these defaults by setting *option_single* to false using the *pop_editoptions* function. The top-level functions in the PREP library automatically set *option_single* to false and consistently maintain double precision computations throughout.

### Referencing

Some EEG headsets (such as Neuroscan) use amplifiers that apply common mode rejection directly, while other headsets (such as Biosemi) require that researchers subtract a reference in post processing to achieve optimal signals. Common choices for a reference signal include the signal at a particular channel, a mastoid channel, the average of two mastoid channels, or the overall signal average. We have found all of these choices to be problematic. For either mastoid or ordinary average referencing, poor contact of an EEG sensor can increase the respective signal variance by several orders of magnitude relative to other channels and thereby contaminate the entire dataset. Removing the average of all of the channels somewhat mitigates this effect, but does not eliminate the problem. The average can be highly skewed by a single outlier.

Using mastoid referencing is problematic for large-scale analysis for several reasons. Many researchers do not record mastoids at all and the actual recording locations may vary across experiments that do record mastoids. In addition, using a mastoid reference introduces a single point of failure—a loose mastoid at any point during the session can introduce enormous artifacts. We also found that average referencing produces data with different statistics than mastoid referencing does. These differences influence the consistency of downstream operations. Several authors (Essl and Rappelsberger, 1998; Hu et al., 2010) have demonstrated the impact that referencing has on bivariate measures such as correlation, phase synchrony, and coherence.

**Figure 3:**
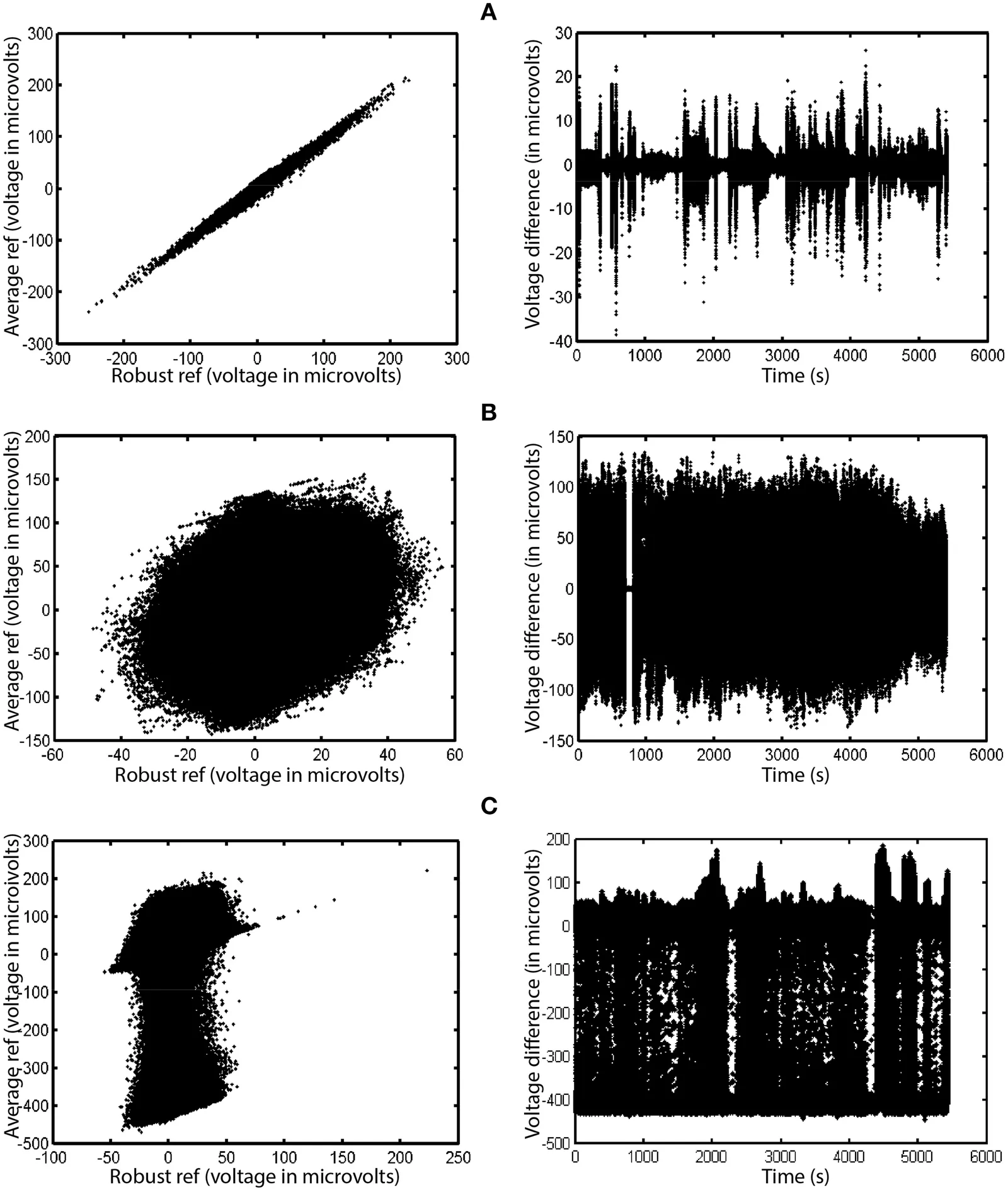
**A comparison of ordinary average reference and robust average referencing in three 32-channel Neuroscan datasets**. Datasets have been high-pass filtered at 1 Hz. Each row corresponds to a different dataset from the same collection. Points on the graph correspond to individual time points in their respective datasets. The left graph in each row shows the ordinary average reference (with no bad channel interpolation) on the vertical axis and the robust average reference on the horizontal axis. The right graph shows the difference between the ordinary and robust average references as a function of time. **(A)** Example where correlation between the two is 0.996. **(B)** Example where correlation is 0.248. **(C)** Example where correlation is 0.171.

To mitigate the interaction between referencing and bad channels, we introduce a robust referencing algorithm. The premise of robust referencing is that noisy channels can irrecoverably contaminate the signal when preprocessing applies average referencing prior to bad channel detection. In order to get a consistent reference and to detect bad channels uniformly, one has to estimate the true average reference when there are no bad channels. Figure 3 shows some examples of the difference between average reference and robust average reference from a collection of 80 EEG datasets acquired from a 32-channel Neuroscan headset. The ordinary average reference is the average of the EEG channels after line noise removal. The robust average reference procedure, described in more detail below, tries to estimate the true average of the EEG channels after removing contamination by bad channels. The left column plots the robust average reference vs. the ordinary average reference. The right column displays the difference between the average reference and the robust average reference as a function of time. If PREP does not detect any bad channels, these two reference signals will be identical. The graph in the left column will be a 45°, and the graph on the right will be a horizontal line. All of the datasets displayed in Figure 3 have been filtered at 1 Hz for display purposes.

Figure 3A shows the results for a dataset whose average and robust average references have a 0.996 correlation. Although the correlation between the two references is high, the reference variability can still be as large as 20% of the signal at different times during the experiment. Ultimately, the PREP pipeline interpolated two channels of this dataset during robust referencing.

Figure 3B shows a dataset whose average and robust reference have a correlation of 0.248. The variability is due to a single, very noisy channel that had intermittent behavior of dropouts and large amplitude variations. Here, robust referencing makes a significant difference throughout the entire dataset.

Figure 3C shows a dataset whose average and robust reference have a correlation of 0.171. Again, the very noisy reference pattern is due to a single intermittent channel. The preprocessing pipeline interpolated a single channel of this dataset after robust referencing.

**Figure 4:**
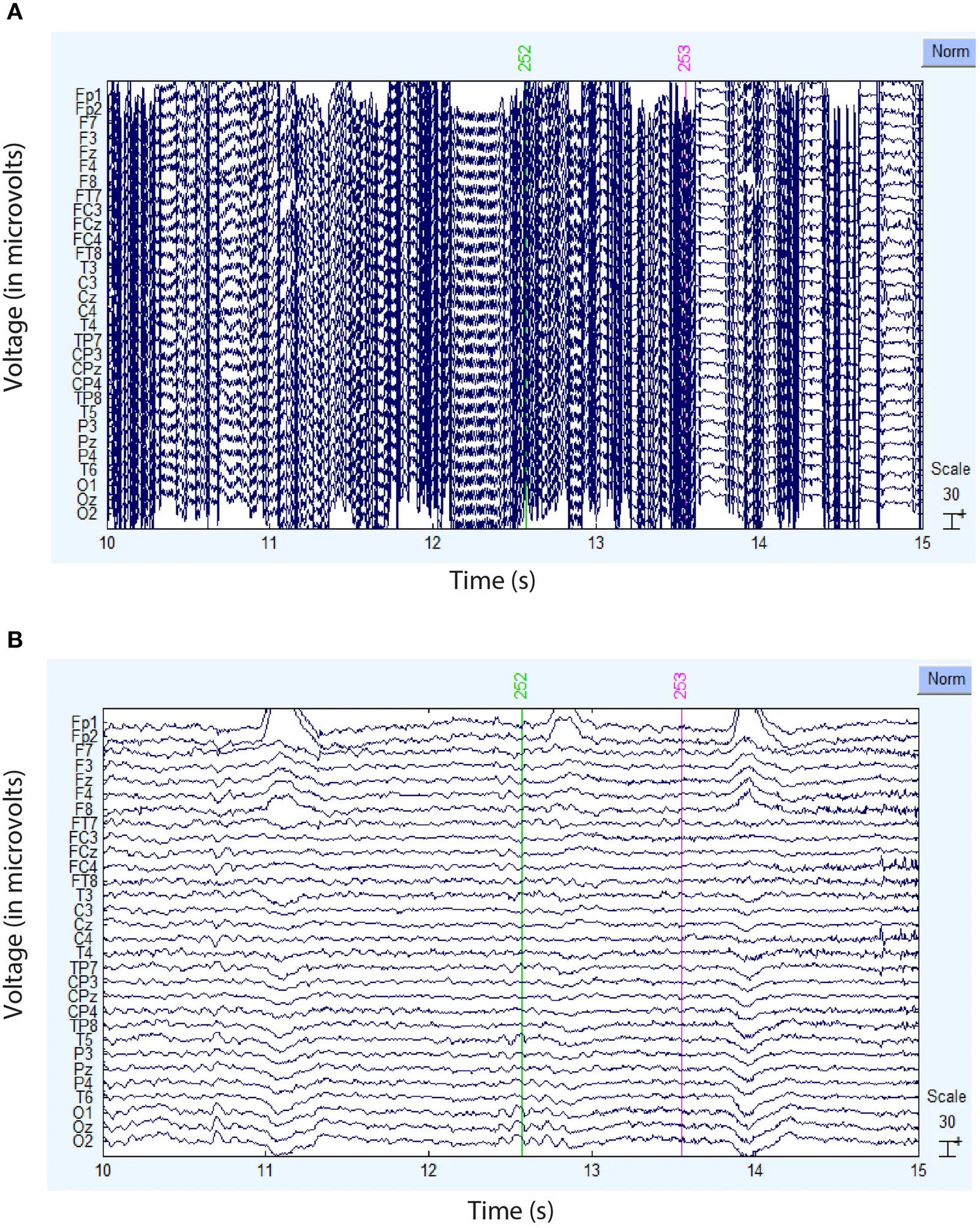
**Comparison of the ordinary and robust average references for the dataset of Figure 3B. (A)** Example signal after ordinary average referencing. **(B)** Same signal using robust average referencing. The data has been high-pass filtered at 1 Hz.

Figure 4 compares the actual signal after average reference (top panel) and robust average reference (bottom panel) for a typical time segment for the dataset shown in Figure 3B. Ultimately, the preprocessing pipeline interpolated four channels of this dataset during robust referencing. The proposed robust referencing approach produces the same results as average referencing if there are no bad channels.

#### Overview of the referencing procedure

The premise of robust referencing is that bad channels should be computed after data has been referenced to a reference signal that is maximally similar to the true average of the signal, i.e., if none of the channels were bad. This approach allows the algorithms to consistently apply thresholds (which are mostly z-score based) without contamination by low recording SNR signals. The algorithm proceeds in two phases: estimate the true signal mean and use the signal referenced by this mean to find the “real” bad channels and interpolate. To summarize the referencing procedure:

#### Phase 1: estimate the true signal mean

EEGTemp = EEG – initial robust estimate of mean (median by default)

badChannels = [];

iterations = 0;

repeat

Detect bad channels for EEGTemp using *findNoisyChannels*
Add any newly detected bad channels to badChannels
*break* from loop if badChannels didn't change or iteration criteria has been met
newMean = mean of EEG with all current badChannels interpolated
EEGTemp = EEG − newMean
iterations = iterations + 1;

end repeat

referenceSignal = mean of EEG with current list of bad channels interpolated

#### Phase 2: find the bad channels relative to true mean and interpolate

EEG = EEG – referenceSignal
Detect bad channels for EEG using *findNoisyChannels*
EEG = EEG with bad channels interpolated in EEG
referenceCorrection = mean EEG
EEG = EEG – referenceCorrection
referenceSignal = referenceSignal + referenceCorrection

For most EEG datasets, the iteration of phase 1 is unnecessary as the algorithm interpolates all bad channels on the first step. However, extremely noisy channels may skew even the initial robust statistics used in the algorithm, and the z scores adjust once the algorithm interpolates extreme channels. The second phase of the algorithm allows “channel forgetting,” since exactly which channels the algorithm accumulates as bad can depend on what the initial estimate of the reference is. Detecting bad channels relative to the “true mean” allows a much more uniform approach to setting thresholds needed for automated processing.

### Detecting noisy or outlier channels

Several stages of the pipeline require the detection of bad or outlier channels. The current version of the noisy channel detector uses four primary measures: extreme amplitudes (*deviation criterion*), lack of correlation with any other channel (*correlation criterion*), lack of predictability by other channels (*predictability criterion*), and unusual high frequency noise (*noisiness criterion*). Several of the criteria use a robust z score, replacing the mean by the median and the standard deviation by the robust standard deviation (0.7413 times the interquartile range). The algorithm also detects channels that contain any *NaN* (not-a-number) data or that have significant periods with constant values or very small values.

The deviation criterion calculates the robust z score of the robust standard deviation for each channel. Channels designated as *bad-by-deviation* have a robust z score greater than 5. This strategy accounts for differences in amplitude across datasets and does not identify channels that capture eye-blinks and most muscle activity as noisy. We also calculate a robust amplitude adjusted z score for each channel in small, non-overlapping time windows (1 s by default), using the overall robust median and the overall robust standard deviation in the z-score calculation. We retain the windowed values for downstream use in the reporting functions.

The correlation criterion is based on the observation that the low frequency portion of EEG signals is somewhat correlated (but not too correlated) among channels. Using signals low-pass filtered at 50 Hz, we calculate the correlation of each channel with the other channels in small, non-overlapping time windows (1 s by default). We calculate the maximum absolute correlation as the 98th percentile of the absolute values of the correlations with the other channels in each window. The algorithm designates a channel as *bad-by-correlation* if this maximum correlation is less than a threshold (0.4 by default) for a certain percentage of the windows (1% by default). We also retain the individual maximum correlations in each window for downstream reporting.

The predictability criterion also relies on the channel correlations of the low frequency portion of EEG signals. Although the *bad-by-correlation* measure effectively detects most bad channels, there are some situations in which channels “go bad together.” We use the RANSAC (random sample consensus) method (Fischler and Bolles, 1981) to select a random subset of (so far) good channels to predict the behavior of each (excluded from subset) channel in small non-overlapping time windows (5 s by default). The implementation is based on functions in BCILAB (Kothe and Makeig, 2013).

The *bad-by-RANSAC* channels behave in a manner poorly predicted by the other channels. Before applying RANSAC, we remove channels designated as noisy by other methods. We then select a random subset of predictor channels for each channel (25% by default). If not enough channels remain to form the required subsets, the algorithm terminates. The RANSAC algorithm uses a method of spherical splines for estimating scalp potential based on algorithms proposed by Perrin et al. (1989). *Bad-by-RANSAC* channels have a correlation less than a threshold (0.75 by default) with their RANSAC-predicted time courses on more than a certain fraction of the windows (0.4 by default). The RANSAC default window size is 4 s.

The noisiness criterion of signal quality uses a robust estimate of the ratio of the power of the high frequency components to the power in the low frequency components. We apply a 50 Hz low pass FIR filter to separate the low and high frequency components. We define the noisiness as the ratio of the median absolute deviation of the high frequency component over the low frequency component for each channel and compute a robust z score relative to all of the channels. Channels designated as *bad-by-HF-noise* have a robust z score greater than 5. As with amplitude, we calculate a robust noise adjusted z score for each channel in small, non-overlapping time windows (1 s by default) and save the windowed z scores for downstream reporting.

The deviation criteria works well for detection of unusually high amplitudes, but does not work well for extremely low, non-zero amplitudes. Furthermore, once PREP subtracts a reference signal, these *low-SNR* channels will essentially contain the channel mean and may no longer have a low amplitude or poor correlation. To handle this case, we designate channels that fail both the *bad-by-correlation* and *bad-by-HF-noise* as *bad-by-lowSNR* channels. Once it detects such a channel, the PREP pipeline puts the channel in the class of unusable channels, such as those containing invalid data values (*bad-by-NaN*) and sections with a constant value (*bad-by-NoData*). PREP removes these channels from consideration and interpolates throughout without having to redetect these channels as bad.

The selection of default parameters in the various detection methods was determined empirically and tested on a variety of different datasets using temporary high pass filtering at 1 Hz. All of the calculations reported here use the default settings.

#### Interpolation of bad channels

The PREP pipeline uses the *spherical* option of EEGLAB *eeg_interp* function for channel interpolation. This function uses Legendre polynomials up through degree 7. To test this choice, we compared this interpolation method with the *v4* option and two other spherical interpolation functions, including the one used in RANSAC. We applied a rank sum test on the correlation of the interpolated channel with the actual channel and found no significant difference between the three spherical interpolation methods. The *v4* option performed significantly worse. The results were not sensitive to whether a block of channels was removed in the immediate vicinity of the bad channel or whether the interpolation was done in ICA space and projected back. As expected correlations for interpolated channels on the cap edges were not as good as those for channels in the interior.

### Computational considerations

Table 1 summarizes the computational costs of various stages in the preprocessing pipeline for a typical dataset. The second and third columns indicate complexity of the algorithm as the number of channels and frames becomes large. As indicated by the table, our implementation is able to take advantage of the significant parallelism present in various stages of the algorithm. The line-noise removal time is essentially linear in the number of channels and windows, but also depends on the number and size of significant frequency peaks. The correlation calculations dominate the robust reference time, but this time also depends on whether the dataset requires multiple iterations of the average reference procedure to remove all noisy channels. We ran the algorithms using MATLAB version 2014a and EEGLAB version 13.4.4b on a Dell Precision T7610 Windows 7 machine with two Xeon 2.6 GHz processors and a total of 12 cores. We obtained a speedup of almost 10 for the line noise portion of the calculation. The speedup for the referencing portion was about 3.5.

**Table 1:** **Parallelism and performance for preprocessing pipeline**.

**Step**	**Channels (*c*)**	**Frames (*n*)**	**Parallelism**	**Time (s) no parfor**	**Time (s) with parfor**
High-pass filter	O(*c*)	O(*n*)	EP by channel	10	10
Line noise	O(*c*)	O(*n*)	EP by channel	492	50
Reference	O(*c^2^*)	O(*n*)	EP by time window	121	32

*Times are for processing a 64-channel Biosemi dataset with 313,856 frames. EP stands for embarrassingly parallel*.

## Reporting and some example results

The specific examples in this paper come from application of the PREP pipeline to a number of collections. One collection (B64) uses a 64-channel Biosemi EEG headset and consists of 18 subjects performing a visual oddball task. This collection was a part of a larger, multi-headset comparison study performed at the Army Research Laboratory in Aberdeen MD (Hairston et al., 2014; Ries et al., 2014). Another collection (N32) uses a 33-channel Neuroscan and consists of 40 subjects in 80 sessions performing a lane-keeping task with and without a motion platform. This collection was contributed from an extensive archive of driving studies performed at the National Chiao Tung University by C-T Lin and his collaborators (Chuang et al., 2012, 2014a,b). Statistics in the summary figures are also included for a task load study (N40) during shooting performed by Kerick and collaborators at the Army Research Laboratory using a 40-channel Neuroscan headset (Kerick et al., 2007). The collection consisted of 9 sessions for each of 14 subjects for 126 datasets. We also analyzed the publicly available 109-subject motor imagery dataset (C64) contributed by Shalk and colleagues to Physionet (Goldberger et al., 2000; Schalk et al., 2004). This collection of 1526 datasets contains 14 sessions for each of 109 subjects using a 64-channel BCI2000 headset.

The PREP pipeline uses bad channel interpolation in a central way. We tried the pipeline on some low-density headset data such as acquired from a 9-channel ABM headset with detectors only on the top of the head, as well as a 14-channel Emotiv headset with detectors only on the forehead. While the statistics looked very reasonable after application of the PREP pipeline, we feel that researchers should proceed with caution when there are not enough channels to cover the head for accurate channel interpolation.

We also applied the pipeline to the public data released for the KaggleBCI competition (“Description—BCI Challenge @ NER 2015 | Kaggle,”^1^). This data consisted of 5 sessions for each of 26 subjects (130 datasets) using 56 passive Ag/AgCl EEG sensors (Margaux et al., 2012). The data was referenced to the nose prior to being released. The PREP pipeline did well on this collection, although some of the overall summary measures (described in the next section) were shifted slightly relative to other collections.

**Figure 5:**
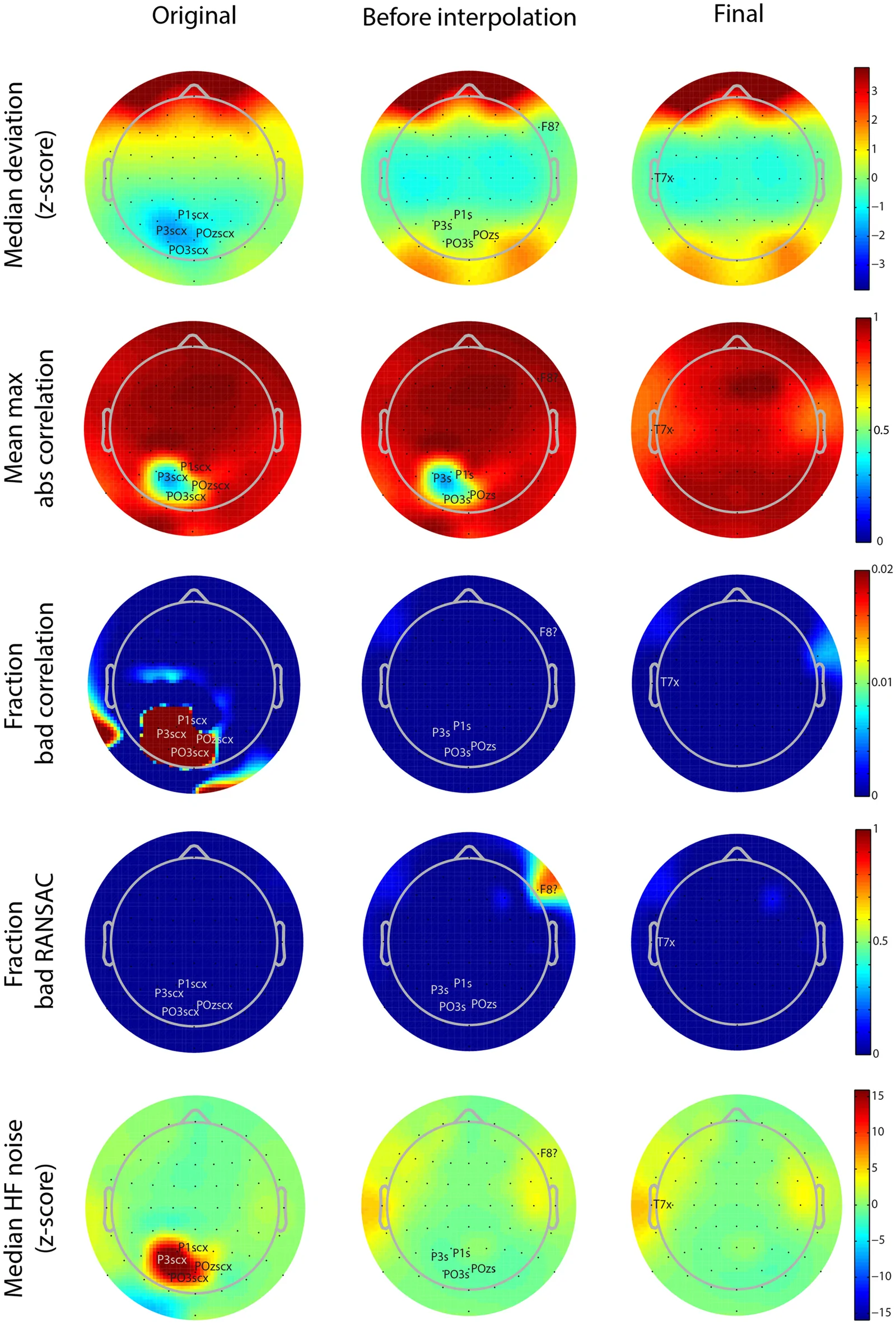
**Scalp map visualizations of noisy channel characteristics for a 64-channel Biosemi dataset produced by the PREP reporting facility**. Each graph indicates channel positions by dots and labels only those channels found to be bad. In each case the channel label is followed by an indicator of cause (c, correlation, x, noise, ?, RANSAC, s, low SNR). The three columns correspond to the three stages in the robust reference procedure (original signal, after reference but before interpolation, and final). Each row corresponds to a different measure (see text). Color scales are consistent across the rows.

**Figure 6:**
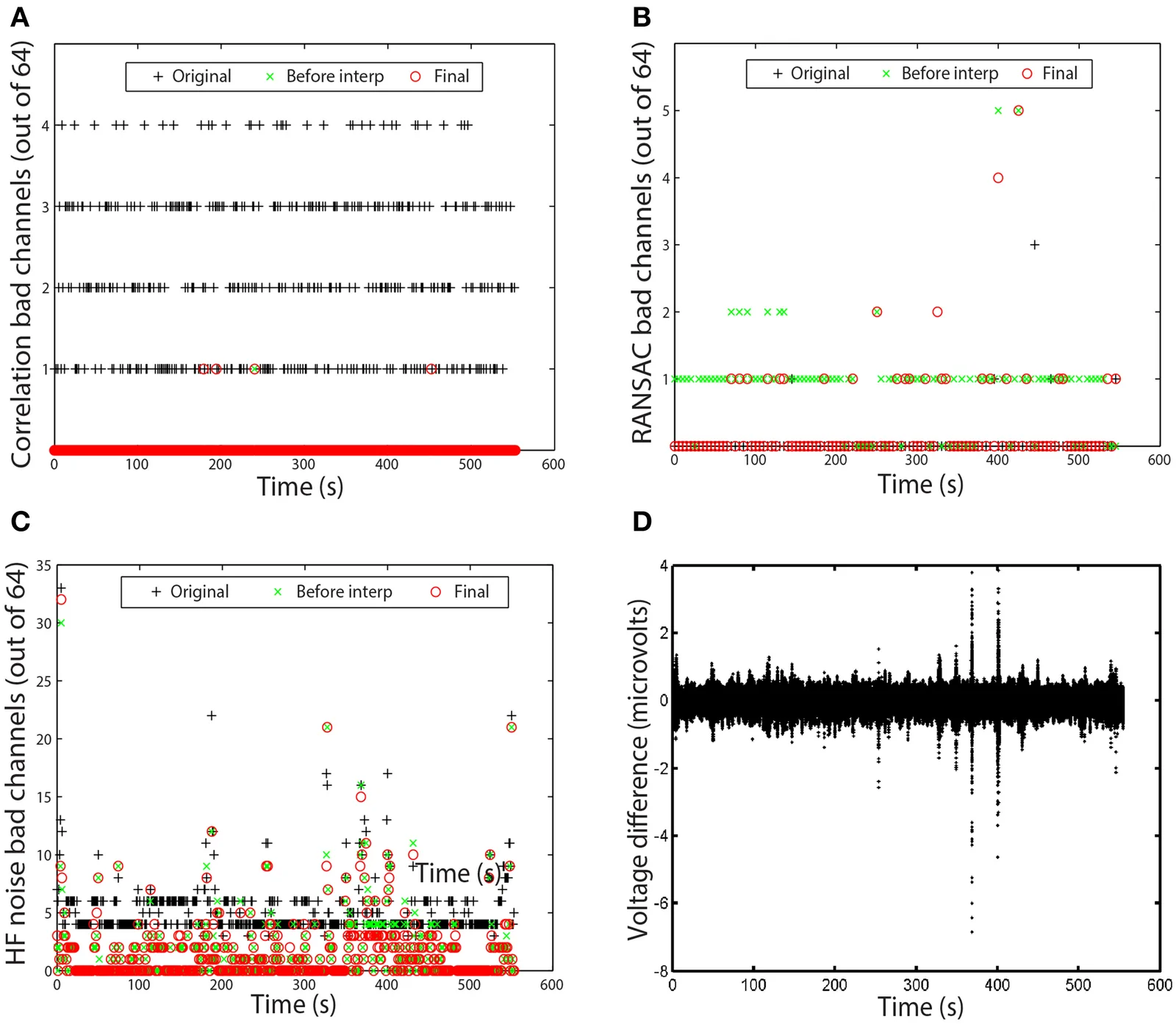
**Examples of time measures available in the PREP reporting facility**. Graphs correspond to the data set of Figure 5. **(A)** Number of channels that failed the channel correlation criterion as a function of time. Each point corresponds to a value computed in a 1-s window. **(B)** Number of channels that failed the RANSAC criterion. Each point corresponds to a value computed in a 4-s window. **(C)** Number of channels that failed the HF noise criterion. Each point corresponds to a value computed in a 1-s window. **(D)** Voltage difference in microvolts between average and robust reference as a function of time. Each point corresponds to a data point in the dataset.

**Figure 7:**
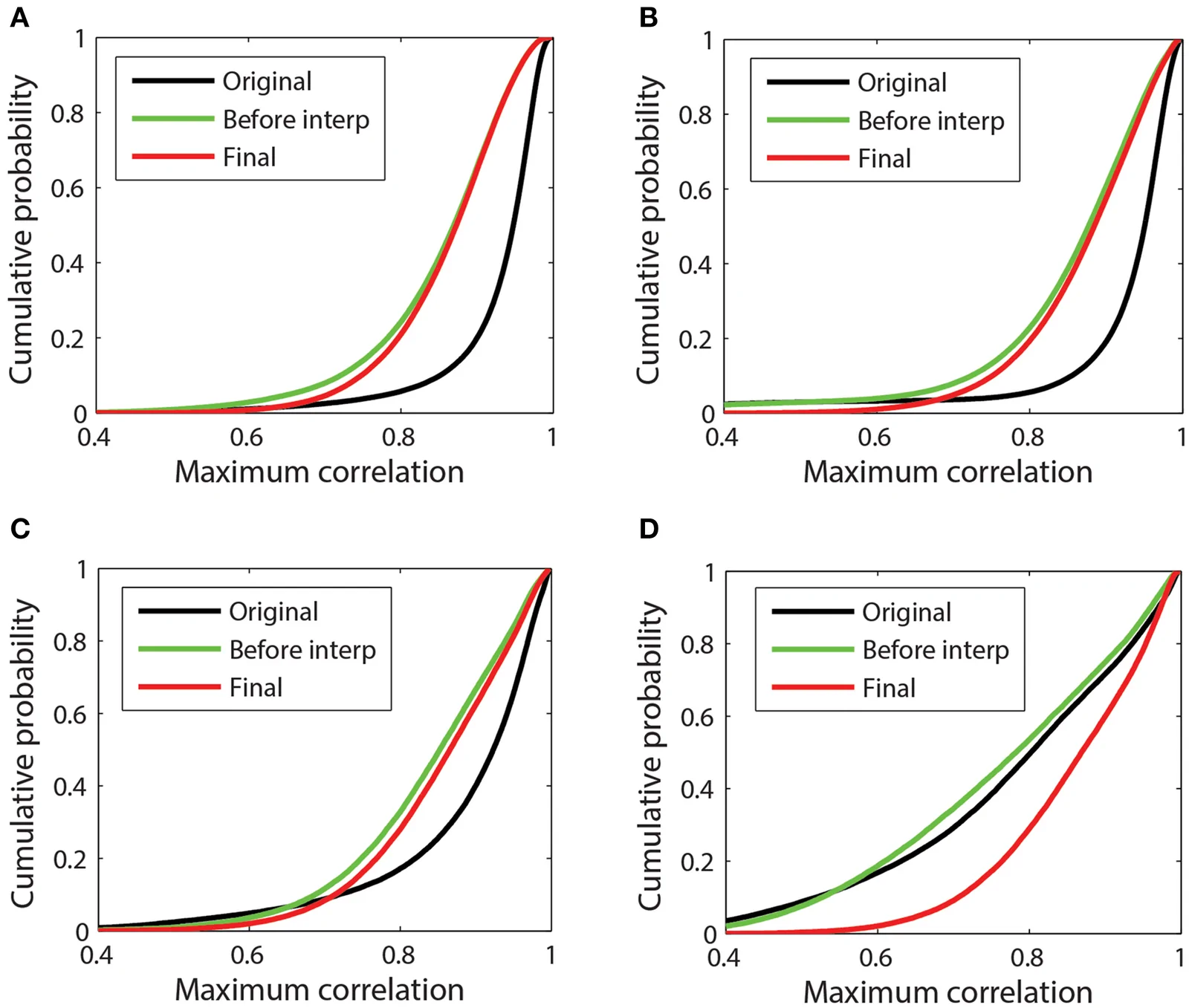
**Cumulative probability distribution of the maximum absolute correlation (i.e., 98th percentile) for each channel with other channels in 1-s time windows. (A)** 32-channel Neuroscan dataset plotted in Figure 3A. This dataset has mean max abs window correlations of [original = 0.93; before interp = 0.85; final = 0.86]. **(B)** 32-channel Neuroscan dataset of Figure 3B. This dataset has mean max abs window correlations of [original = 0.92; before interp = 0.86; final = 0.88]. **(C)** 64-channel Biosemi dataset of Figures 1B, 4. This dataset has mean max abs window correlations of [original = 0.89; before interp = 0.85; final = 0.86] **(D)** A 64-channel Biosemi dataset with a large number of bad channels due to low correlation. This dataset has mean max abs window correlations of [original = 0.79; before interp = 0.78; final = 0.87].

The optional *prepPipelineReport*, which is designed to be run after *prepPipeline*, produces a summary of statistics and visualizations at each step. The reporting functions create an HTML summary file that has a brief synopsis of the results for each dataset and a link to the more detailed report. Figures 3, 5–**7** are from this report. At the end of a preprocessing run, the pipeline stores the status information in the *EEG.etc.noiseDetection* structure of the EEGLAB *EEG* structure. The *noiseDetection* structure includes the reference signal, a list of the channels identified as bad as well as the window statistics for the bad channel measures. These window statistics allow users to detect anomalous behavior in small time windows, which can be useful in identifying bad epochs. The structure also retains the original signals for interpolated channels, so that users can choose not to interpolate in particular epochs downstream.

The preprocessing suite also has functions for extracting the statistics from an entire collection as well as visualization functions and issue reports listing datasets that are likely to be problematic after referencing. In this way, a user can quickly scan for issues in very large collections. We extracted the figures in this section from the individual data reports.

Functions are also provided in a separate toolbox (“VisLab/EEG-HDF5-Tools,”^2^) to store the results in an HDF5 file (“The HDF Group—Information, Support, and Software,”^3^) that is readable by any tool that supports HDF5. EEG-HDF5-Tools package includes source functions to read these files in MATLAB, R, Python, and C. A researcher can include the appropriate source library in their own applications to read the data and metadata in these formats. The referencing procedure depends critically on the detection of bad or noisy channels. As explained in Section Detecting Noisy or Outlier Channels, we use four main criteria: deviation, correlation, predictability, and noisiness.

As mentioned above, PREP produces a PDF report detailing the results of referencing and bad channel detection. These reports allow users to see at a glance where the issues are in the dataset. Figure 5 shows the different scalp maps produced during the automatic reporting. The columns represent the three collection points for statistics retained in the *EEG.etc.noiseDetection.reference* structure: the original data, the data after referencing but before bad channel interpolation, and the final version of the data after interpolation and referencing. The rows of the figure correspond to different quantities that PREP reports. Each scalp map labels the relevant bad channels for that point annotated by the cause of the issue (*NaN* = “n,” *No data* = “z,” *Dropout* = “d,” *Correlation* = “c,” *Deviation* = “+,” *Predictability* = “?,” *Noisiness* = “x,” *low SNR* = “s”). PREP uses the same color scaling for all maps in a row, so that users can compare them.

All of the graphs from Figures 5, 6 come from the same 64-channel Biosemi dataset. The first row shows the median over 1-s windows of the z-scores of the robust channel deviation. Because the deviation is always positive, it is usually not possible to obtain large negative z scores. However, this dataset shows a patch of four posterior channels with very low deviation. The signals from these channels appear flat relative to the other channels when viewed on a scroll plot. While these channels do not fail the deviation criterion (|z-score| > 5), they fail both the correlation (c) and noisiness criteria (x). PREP identifies these channels as having low SNR (s) and designates them as unusable. In downstream iterations, PREP sets their deviation z score to zero, so they appear in green, but designates these channels as low SNR (s).

The second row of Figure 5 shows the average of the largest (98-th percentile) absolute correlation of each channel with any other channel in 1-s windows. When channel signals contain a large common additive noise component, this value will be very close to 1.0. However, the four noisy channels have a much lower maximum correlation with any other channel.

The *bad-by-correlation* criterion requires that the channel have a low correlation in a certain percentage of the windows (1% for PREP defaults). The third row of Figure 5 shows the percentage of windows in which the channel was *bad-by-correlation*. The color scale goes from dark blue for 0% and saturates at deep red at 2% bad. The first map in row shows that the four channels are bad for at least 2% of the windows. The two red areas on the rim of the map are an artifact of the extrapolation used for the color map and do not contain any channels.

The fourth row of Figure 5 shows the fraction of time that RANSAC projections poorly predict channel signals. Once PREP removes the reference along with common additive noise, such unpredictability often comes more distinguishable. RANSAC fails to predict channel F8 about 70% of the time. PREP adds this channel to its interpolation list for the final interpolation. Note that interpolation tends to represent channels on the edge of the cap less accurately than interior channels, and so PREP tends to detect borderline edge channels as bad more frequently than other channels. However, 70% is a very large percentage and unlikely to represent a borderline event.

The final row represents the z score of the ratio of high frequency power (>50 Hz) and low frequency power (1-50 Hz). The four channels with low SNR have a noisiness z score of around 15. In the final stage, PREP classifies the T7 edge channel as noisy. However, as the map color shows, this channel has a noisiness z score very close to the borderline cutoff of 5.0.

In most cases, no bad channels remain after the PREP pipeline has processed the data. However, occasionally an edge channel will be on the borderline, usually through the correlation or noisiness criterion. PREP provides additional visualizations of the specific windows in which channels are bad to help researchers understand the nature of these issues. Figures 6A–C present examples of these visualizations. Each visualization shows each window using a point. A + represents the original signals (column 1 of Figure 5), a × represents the signals before interpolation but after referencing (column 2 of Figure 5), and an o represents the final signal (column 3 of Figure 5). The vertical axis displays the number of channels that fail the particular criterion. Each point on these graphs represents a window.

Figure 6A shows the *bad-by-correlation* criterion. Most windows have one or more channels failing the correlation criterion. Figure 6B shows the *bad-by-ransac* criteria. We see that T7 fails RANSAC in almost all windows before interpolation (the green ×s). Figure 6C shows the bad-by-HF-noise criterion. At least four channels fail in nearly every window in the original signal. However, four channels (P1, P3, POz, PO3) fail both noisiness and correlation in the original signal, PREP designates them as unusable and removes them from further consideration. Hence, they don't appear in the plots as bad. The correlation and noisiness criteria use 1-s windows, while RANSAC uses 4-s windows. Hence, Figure 6B appears to have fewer points than Figures 6A,C.

Figure 6D shows the time course of the difference between the ordinary average reference with no channel interpolation and the robust reference with channel interpolation. This shows a very small difference between the two, indicating that the dataset is relatively clean and does not have major issues. This result is in clear contrast to the examples of the right column of Figure 3.

Referencing generally removes common additive noise, which usually reduces the overall maximum channel correlations. Figure 7 presents a more detailed look at the cumulative distributions of channel correlations before referencing, after referencing but before interpolation, and after final interpolation for four example datasets. The distribution consists of the values of the 98th percentile of the maximum absolute correlation for each channel in each window. Figure 7A corresponds to the 32-channel Neuroscan dataset whose references were plotted in Figure 3A. This dataset is relatively clean. Although PREP interpolated two channels, the correlation between the ordinary average reference and the robust average reference was 0.995. Average referencing reduces the mean of the distribution from 0.93 to 0.86. After interpolation the mean increases slightly to 0.88. This dataset had already been mastoid referenced prior to submission to our repository.

Figures 7B,C correspond to the 32-channel Neuroscan dataset of Figure 3B and the 64-channel Biosemi dataset of Figure 1B, respectively. Both datasets exhibit some low-correlation channel windows before referencing, but referencing brings the cumulative distributions in line with the cleaner data of Figure 7A. Both of these datasets had a single very bad channel whose low correlations with other channels is reflected by the left tail in the data before referencing. Figure 7D corresponds to a 64-channel Biosemi dataset that had 19 bad channels distributed over the headset. The mean of the distribution before any referencing is 0.79. After referencing but before interpolation, the mean is 0.78. After bad channel interpolation, the mean becomes 0.87 and the cumulative correlation of this dataset was virtually indistinguishable from the others.

It should also be emphasized that the robust referencing does not deal with any of the subject-generated artifacts such as muscle and eye movements. Bad epoch removal is not part of the referencing procedure. However, the reporting mechanism keeps statistics over 1-s windows and researchers can easily use these statistics to flag potentially bad segments.

## Summary measures

One goal of the PREP pipeline is to produce quick summary measures that allow analysts to determine quickly whether a particular dataset might have issues. We have developed several useful heuristics for detecting issues in the data. We provide individual dataset and collection summaries that allow researchers to pinpoint issues in large datasets and dataset collections. The *runStatistics* reads a collection of processed datasets and creates a statistics structure and an issues report. The criteria we currently use to flag datasets with serious issues include:

- Robust referencing does not move the mean of the median max channel correlation closer to [0.80, 0.91] if not initially in the interval.
- The mean of the max channel correlation is above 0.91 and the median of the max channel correlation is greater than 0.95 after robust referencing.
- Robust referencing interpolated more than 25% of the reference channels.

**Figure 8:**
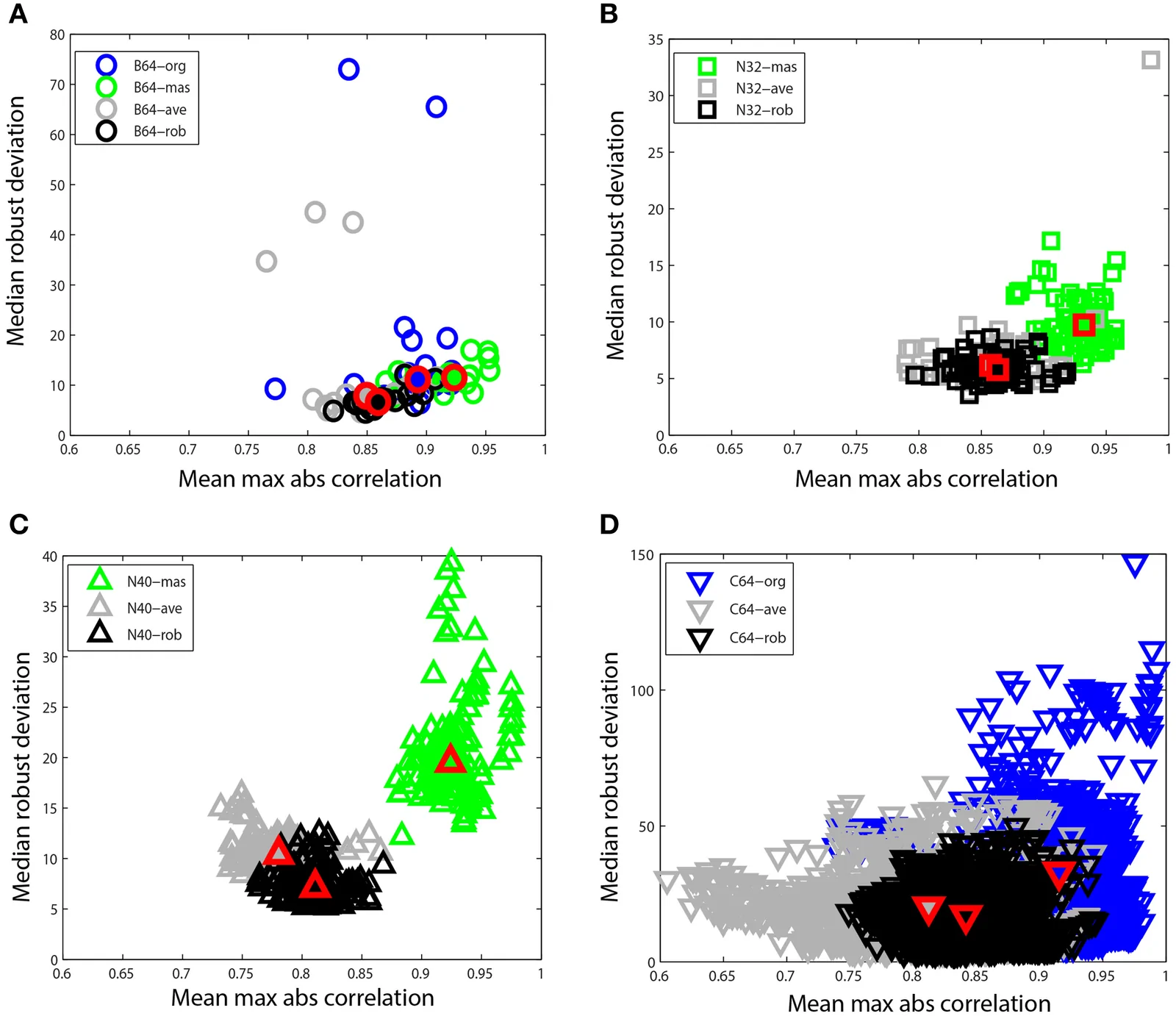
**Mean max absolute window correlation vs. median robust window channel deviation for different referencing schemes**. Each unfilled point represents a data set. Blue points correspond to data that was originally unreferenced or whose original reference status is unknown. Green points correspond to mastoid referenced datasets, and gray points correspond to average referenced datasets. Black points correspond to datasets after robust referencing with channel interpolation. The large solid shapes outlined in red represent overall medians for the corresponding data collections. **(A)** B64 is an 18 subject 64-channel dataset (circles). **(B)** N32 is an 80 session 40 subject driving simulation using a 32-channel Neuroscan headset (squares). **(C)** N40 is a 14-subject 126 session data collection acquired using a 40-channel Neuroscan headset (up triangles). **(D)** C64 is a 109-subject 1526 session data collection acquired using a 64-channel BCI2000 headset (down triangles).

**Figure 9:**
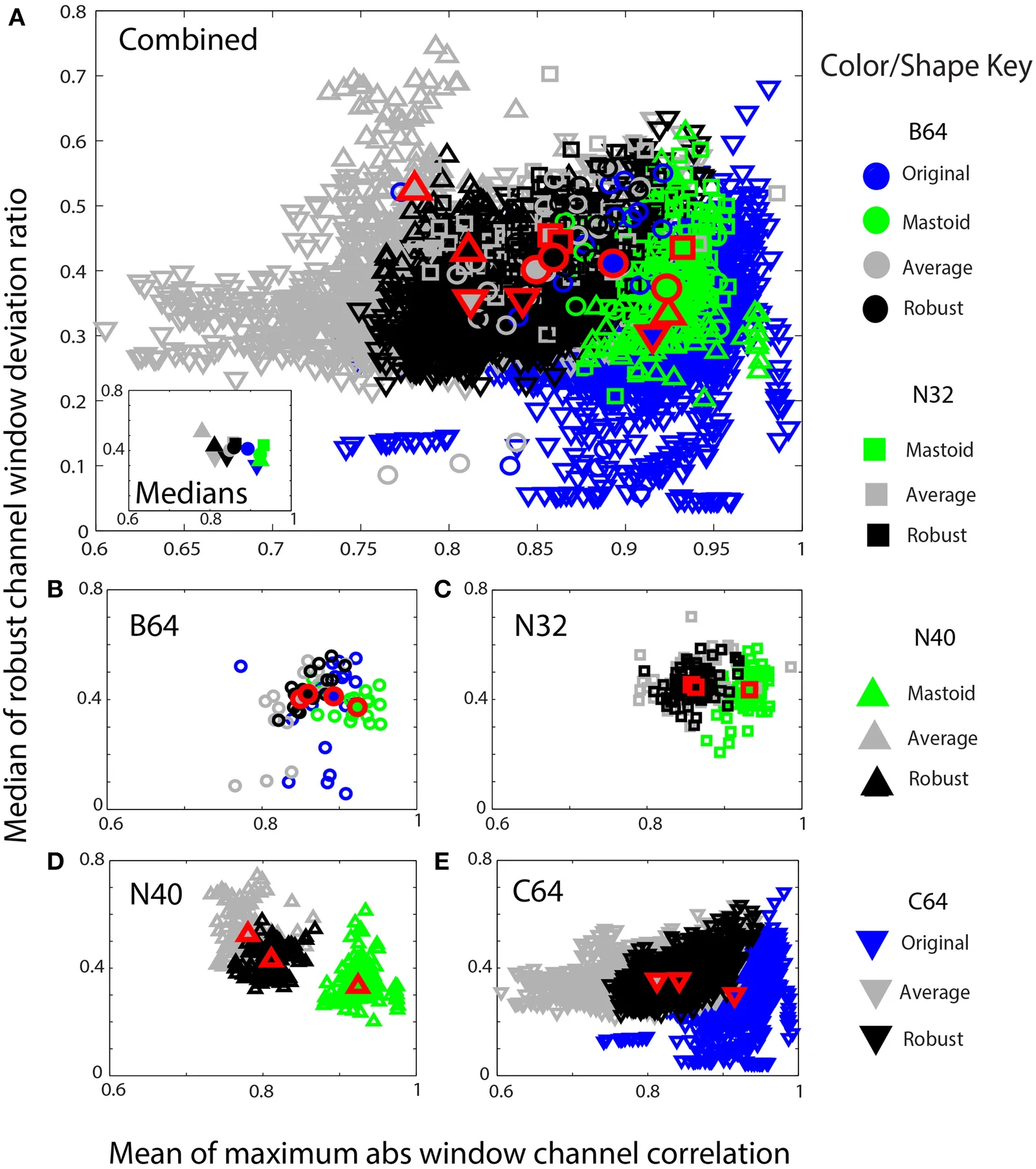
**Mean max absolute window correlation vs. the median of the robust window deviation ratio for four collections**. Each unfilled symbol represents a data set in a collection. The collections are distinguished by shape: circle (B64) square (N32), up triangle (N40), and down triangle (C64). The corresponding filled symbols, also outlined in red, give the median of the statistic for that collection. Blue points represent data that was originally unreferenced or whose reference is unknown. Green points represent mastoid referenced datasets, and gray points represent average referenced datasets. Black points represent robust referenced datasets with channel interpolation. **(A)** An overplotting of all datasets. The inset displays the collection medians. **(B)** B64 is an 18 subject 64-channel dataset. **(C)** N32 is an 80 session 40 subject driving simulation using a 32-channel Neuroscan headset. **(D)** N40 is a 14-subject 126 session data collection acquired using a 40-channel Neuroscan headset. **(E)** C64 is a 109-subject 1526 session data collection acquired using a 64-channel BCI2000 headset.

The *createCollectionStatistics* function takes a list of files as input and produces a statistical collection summary. The *showNoisyStatitics* function produces summary visualizations for the collection from the statistical summary. The *runCollectionComparison* script compares collection summary statistics across collections and produces summary graphics similar to those of Figures 8, 9.

PREP provides several other functions to show additional visualizations of the collection as a whole and in the context of other collections. Two measures used throughout are the *maximum absolute correlation* and the *robust window deviation*. Both are window measures. If a dataset consists of *c* channels and *w* windows (1-s by default), both measures are *c* × *w* arrays. The maximum absolute correlation holds the maximum absolute value of the correlation of each channel with other channels in the window, while the robust window deviation is 0.7413 times the interquartile range of the channel in the window. Two summary measures of the dataset are the overall mean of the maximum absolute correlation and the overall median of the robust window deviation.

Figure 8 compares these two summary measures for different referencing schemes for four of the data collections described at the beginning of Section Reporting and Some Example Results. Figure 8A shows B64, which included mastoid channels and was initially unreferenced (B64-org). We referenced this collection by averaging the mastoids (B64-mas) and by calculating the average of the EEG channels (B64-ave). We compared this to the result of the robust referencing procedure (B64-rob). Each point on the graph represents a dataset with a different referencing technique applied. The mastoid channels for this data collection were relatively clean and gave robust deviations that were similar to those of robust referencing. However, both the original and the average reference had some significant outliers. The maximum absolute correlation for mastoid referencing was higher than for robust referencing.

Figure 8B shows N32, which was referenced to the mastoids during acquisition. The mastoid references show a distinctly higher maximum correlation and the robust deviations are more widely spread. Figure 8C shows N40, the shooter collection that was referenced to one of the mastoids during acquisition. This data collection was more noisy than those datasets acquired under more controlled laboratory conditions. The robust reference values are nicely clustered, while the deviations for mastoid referenced datasets are widely spread. Both N32 and N40 were referenced during acquisition, so unreferenced original data wasn't available for comparison.

Figure 8D shows the C64 motor imagery collection available on Physionet. The documentation for this dataset did not specify the referencing status of the raw data, so we label the data as C64-org. However, the statistics are consistent with mastoid referencing. There is a considerable spread in both average referencing (C64-ave) and the original referencing (C64-org) when compared with robust referencing (C64-rob). Compare the scales on the four plots.

Figure 9 shows similar information in a scaled format for comparison across the collections. The shapes correspond to specific data collections, while the colors correspond to the type of reference. The horizontal axes show the mean maximum channel correlation as in Figure 8. The vertical axis shows the robust channel deviation ratio. If D is a *c* × *w* array containing the 0.7413 times the interquartile ranges of data in *w* windows for *c* channels, the robust deviation ratio is the robust standard deviation of D divided by the median of D. Figure 9 represents each data set by the overall median of this ratio.

Figure 9A shows a composite of all of the datasets from the four collections using the same color and plotting scheme as in Figure 8. The inset in Figure 9A shows the overall collection medians. Figures 9B–E show the individual plots for B64, N32, N40, and C64, respectively. The robust referencing (shown in black) is better clustered with fewer outliers and overlaps well among the four collections.

To summarize:

- Mastoid referencing has very different statistics than either average referencing or robust referencing. The mean max window correlation is generally higher and the SDR/MED ratios of deviation are generally lower in mastoid referencing.
- Robust referencing and averaging referencing have similar statistics, provided that there are no extremely bad channels.
- Comparisons of robust referencing across collections show no statistical differences in the correlation or ratio measure and some difference in channel deviation measure.
- Performing a mastoid reference initially and then later re-referencing using either average or robust reference produces essentially the same results as if no mastoid referencing had been done.
- The order of performing mastoid referencing relative to filtering and line noise removal makes essentially no difference.

Again, we emphasize that these statements only hold true when all arithmetic is done in double precision with no intervening conversions.

## Other tests

### Tests of bad channel detection with synthetic data

In order to evaluate the accuracy of the noisy channel detection procedure that underpins the robust reference, we applied *findNoisyChannels* to datasets with synthetically generated noisy channels. To generate noisy channels, we selected raw data from five sessions of the 32-channel Neuroscan collection, high-pass filtered at 1 Hz and removed line noise. These datasets had no visibly noisy channels, and the noisy channel detection algorithm did not flag any bad channels. We then introduced a number of noisy channels into each dataset using the following methods:

- Adding Gaussian noise with an amplitude 8 times channel standard deviation.
- Lowering (to one-tenth) amplitude, simulating a weak electrical connection.
- Temporally shuffling the data and making the channel maximally uncorrelated with other channels while having the same amplitude.
- Replacing the data in two channels of the session by amplitude-normalized copies of a single, highest amplitude (often eye or muscle-dominated), channel selected from another dataset repeated or trimmed to the length of the current session. This made the two channels fully correlated, simulating the worst-case scenario where an external noise source produces highly correlated noisy channels with no change in signal amplitude.

Table 2 shows the number of channels containing different types of synthetic noise.

**Table 2:** **Number of channels containing different types of synthetically-generated noise for noisy channel detection tests**.

**Session**	**Noisy channels**	**Gaussian noise**	**Low amplitude**	**Temporally shuffled**	**Fully correlated noise**
5	7	3	1	1	2
14	7	3	1	1	2
30	8	4	1	1	2
31	10	5	1	2	2
68	10	5	1	2	2

*All datasets had 32 channels and were taken from the N32 collection*.

We then applied our noisy channel detection to the modified data from these five sessions after the data had been high-passed filtered at 1 Hz and line noise removed. Table 3 shows a comparison of the channels detected by the pipeline as noisy to the simulated ground truth. The *findNoisyChannels* detection function had a high (81%) sensitivity [true positive rate = TP/(TP + NP)] and a high (97%) specificity [true negative rate = TN/(TN + FP)].

**Table 3:** **Comparison between detected and simulated ground-truth noisy channels out of 160 channels total**.

**Session**	**Noisy channels**	**True positive (TP)**	**False positive (FP)**	**True negative (TN)**	**False negative (FN)**
5	7	5	0	25	2
14	7	5	0	25	2
30	8	8	0	24	0
31	10	8	2	20	2
68	10	8	1	21	2
**Total**	42	34	3	115	8

We also compared the detection performance of PREP with *pop_rejchan* function available in EEGLAB package. We individually tried all three measures for noisy channel detection available for this function (Kurtosis, Probability, and Spectrum) as shown in Table 4. In all cases this function did not detect most of simulated ground truth noisy channels and had a poor (7% or less) sensitivity (compared to 81% or higher for PREP).

**Table 4:** **Comparison between noisy channel detection performance of EEGLAB** ***pop_rejchan*** **with different options and the detection performance of PREP pipeline**.

**Detection method**	**True positive (TP)**	**False positive (FP)**	**True negative (TN)**	**False negative (FN)**	**Sensitivity**	**Specificity**
PREP	34	3	115	8	81%	97%
*pop_rejchan* (kurtosis)	0	5	113	42	0%	96%
*pop_rejchan* (probability)	3	0	118	39	7%	100%
*pop_rejchan* (spectrum)	0	0	0	42	0%	100%

### Effect of pipeline on downstream calculations

To test the effect of filtering and referencing on downstream applications, we looked two simple classification problems: single-subject (SS) classification and leave-one-subject-out (LOSO) classification for the 18-subject 64-channel Biosemi data collection described above. The experiment uses a visual oddball paradigm. The datasets consist of 30–35 target events and roughly seven times the number of non-target events. The datasets were epoched into 1-s epochs.

For these tests, we used two well-known classifiers: linear discriminant analysis (LDA) and hierarchical discriminant analysis (HDCA) (Marathe et al., 2014). For LDA, the features consisted of vectors of length 64 × 512 = 32,768. LDA + PCA uses PCA on each subject to reduce the feature vector dimension to 50. HDCA divides an epoch into sub windows of 1/8 s, resulting in feature vectors of size 64 × 64. HDCA-PCA reduces the channel numbers to 45 from 64 in order to remove linear dependencies between channels due to interpolated channels. Thus, the feature vectors are 45 × 64 = 2880.

The SS classification tests randomly select 30 non-target and 30 target events and run the classification test with 54 training samples and six test samples. The results were the average of repeating the process 50 times.

The LOSO voting classification test uses each of the 18 subjects as the test subject. For each test subject, we built classifiers from the other 17 subjects using balanced training sets of 60 randomly drawn samples (30 samples from each class). The test set from each subject also consisted of 60 samples (30 samples from each class) at random. Each test sample was classified by majority vote of the classifiers built from the other subjects. The tests are the average of 10 repeats.

Table 5 shows the results of the tests. The table indicates the complicated relationships between filtering, referencing, and dimension reduction. Referencing generally improves the results. For example, the AUC for SS LDA goes from 56.3 to 66.0 for robust referencing with a 1 Hz high pass filter. The AUC goes from 69.6 to 71.3 for LOSO. The results for 0.3 Hz high-pass filtering are similar, but the AUCs are generally lower.

**Table 5:** **Effect of referencing, filtering, and dimension reduction on classification accuracy for the 18-subject 64-channel Biosemi visual oddball experiment**.

**Signal type**	**Classifier**							
	**LDA**		**LDA + PCA (50 dim.)**		**HDCA**		**HDCA + PCA (45 ch.)**	
	**SS**	**LOSO**	**SS**	**LOSO**	**SS**	**LOSO**	**SS**	**LOSO**
HP 0.3 Hz	56.8	68.5	86.8	74.6	75.5	71.6	75.8	77.9
HP 1.0 Hz	56.3	69.6	87.3	75.7	90.7	82.2	91.1	84.6
HP 0.3 Hz + RR	63.5	69.5	89.3	78.1	fail	fail	78.5	79.3
HP 1.0 Hz + RR	66.0	71.3	90.5	79.1	fail	fail	91.7	86.5

*Values are reported as AUC. SS, single subject classification; LOSO, leave-one-subject-out classification; RR, robust referencing with channel interpolation*.

HDCA also shows some improvement with robust referencing, particularly for LOSO. However, HDCA appears to be very sensitive to the selection of high-pass filter cutoff, doing much better for a high-pass cutoff of 1 Hz than a high-pass cutoff of 0.3 Hz. HDCA fails for robust referencing without PCA to remove linearly dependent channels. Users should be aware of this when applying algorithms that require matrices to be of full rank.

The results illustrate the importance of testing algorithms with different filtering strategies and of not committing to a filtering cutoff during preprocessing.

A concern about average/robust referencing for P300 analysis is that it removes a portion of the signal common to all channels that is not noise, but neural signal. To test this, we applied LDA with PCA reduction to dimension 20 to the robust reference signal and to the average of the mastoids in the unreferenced signal. We found that SS LDA classification on the above data collection using only the robust reference signal as a feature gives an AUC of 77.9 when the signal is filtered at 0.3 or at 1 Hz. This result indicates that although it may be noisy, the reference signal contains information about the task and may be useful as an additional feature in downstream machine learning tasks.

There is also a commonly held belief that, because their placement, mastoids do not contain as much neural signal and therefore better capture additive common noise. To test this idea, we applied LDA after PCA (dimension 20) to the single time series that is the average of the two mastoids. The mastoid signals high-pass filtered gave a single subject AUC of 82.6 when filtered at 0.3 Hz and 82.4 when filtered at 1 Hz. The results from Table 5 did not include mastoid channels.

As the results of Table 5 show, filtering and referencing can affect downstream analysis in unpredictable ways. HDCA is particularly sensitive to the filtering frequency both in SS and across-subject classification. The results also show that the mastoid electrodes carry important information, as does the reference signal. This particular data collection had very clean mastoid recordings. However, for large-scale cross-collection analysis, mastoid references may not be available or may be unreliable. The PREP pipeline stores the robust average reference of the raw signal in the EEG structure in the field *EEG.etc.noiseDetection.referenceSignal* as part of its reporting. Users can add the signal back in and reference to the mastoids or use the information for feature augmentation if desired. However, as shown by Figures 8, 9, it is important to use a consistent referencing method when doing multi-collection analysis.

## Discussion

Raw data often requires extensive curation and identification in order to be usable for analysis. In addition, preprocessing steps can be quite computationally intensive. A key step for mining EEG across large collections is to develop a standardized preprocessing pipeline that allows researchers to perform a variety of analyses without reference to the raw data. Such a pipeline can provide the basis for sharing data across laboratories and could be the starting point for EEG repository and API efforts such as HeadIT (headit.org), COINS (Scott et al., 2011), GNode (Sobolev et al., 2014), and EEGBase (Moučekk et al., 2014).

EEG preprocessing has generally focused on bad channel/epoch identification and removal. Methods/pipelines for EEG preprocessing often assume referencing and high pass filtering as a starting point for processing and seldom document these early steps in any detail. This work has demonstrated that systematic early-stage identification and interpolation of channels with “non-EEG” behavior is important for normalizing EEG statistics across data collections. Processing methods that interpolate channels should keep a record of these channels with the data, since many downstream analysis algorithms assume that the channel data is linearly independent. The PREP pipeline keeps a complete record of the interpolation, algorithm parameters, and signal statistical characteristics in the *EEG.etc.noiseDetection* structure and stores this structure as a separate HDF5 file for use by other tools. One key design decision for PREP was to defer the selection of high-pass filter to downstream users in order to maximize the usefulness of PREP'ed data across applications.

A number of pipeline-type toolboxes for artifact detection and removal are available as EEGLAB plugins, each addressing a particular preprocessing aspect. ADJUST (Mognon et al., 2011) uses ICA to remove features associated with various stereotypical artifacts, particularly eye blinks, eye movements, and discontinuities. The FASTER pipeline (Nolan et al., 2010) uses a combination of statistical thresholding and ICA to identify and remove contaminated channels and bad epochs, particularly those associated with eye movements, muscle artifacts, trends, and white noise. FASTER is optimized for ERP analyses. TAPEEG (Hatz et al., 2015) combines several automated procedures from Fieldtrip and FASTER with additional special-purpose functions in order to detect bad channels, bad epochs, and bad ICA activations. DETECT (Lawhern et al., 2013) uses machine-learning techniques based on auto-regressive features to identify artifacts of various types, including eye movements, jaw clenching and other muscle artifacts. Safieddine et al. (2012) have proposed muscle artifact removal techniques based on ICA and on EMD (empirical mode decomposition). Kothe et al. have proposed Artifact Subspace Reconstruction (ASR), a method that removes high-variance artifacts from a dataset by comparison to a relatively artifact-free data segment (Mullen et al., 2013). ASR is capable of running online in real time.

The difficulty with adopting any of the artifact removal pipelines as a preprocessing standard is that each makes processing decisions that may preclude use of the data across the full spectrum of applications. The goal of PREP is to perform just enough preprocessing to make the data usable for automated processing by normalizing data statistics across collections. PREP accomplishes this task by a careful application of line noise removal, bad channel detection, and referencing. PREP defers high-pass filtering to downstream processes because filtering is a low-cost operation, and many analyses are sensitive to the exact choice of filters. The data resulting from the PREP pipeline shows more uniform statistical behavior across a variety of headsets and experimental paradigms. Thus, PREP data provides a solid foundation for building large-scale EEG repositories. Users can download the PREP pipeline as freely-available MATLAB library from https://github.com/VisLab/EEG-Clean-Tools. Users should also have the MATLAB Signal Processing Toolbox.

### Data availability statement

Users can download the PREP pipeline as a freely available MATLAB library from https://github.com/VisLab/EEG-Clean-Tools. Users can cite all versions by using the DOI 10.5281/zenodo.22983431.

### Conflict of interest statement

The authors declare that the research was conducted in the absence of any commercial or financial relationships that could be construed as a potential conflict of interest.

## Footnotes

^1^Description—BCI Challenge @ NER 2015 | Kaggle., (n.d.). Retrieved January 11, 2015, from https://www.kaggle.com/c/inria-bci-challenge

^2^VisLab/EEG-HDF5-Tools., (n.d.). Retrieved February 5, 2015, from https://github.com/VisLab/EEG-HDF5-Tools

^3^The HDF Group—Information, Support, and Software., (n.d.). Retrieved December 29, 2014, from http://www.hdfgroup.org.
