# Correction: The PREP pipeline: standardized preprocessing for large-scale EEG analysis

**DOI:** 10.3389/fninf.2026.2015651

**Published:** 2026-10-12

**Authors:** Nima Bigdely-Shamlo, Tim Mullen, Christian Kothe, Kyung-Min Su, Kay A. Robbins

**Affiliations:** 1 Syntrogi Inc., San Diego, CA, United States; 2 Swartz Center for Computational Neuroscience, University of California San Diego, La Jolla, CA, United States; 3 Department of Computer Science, University of Texas at San Antonio, San Antonio, TX, United States

**Keywords:** EEG, artifact, preprocessing, EEGLAB, BCILAB, machine learning, big data

In the published article, in the **Abstract** and the **Discussion**, paragraph 4, the study code is referred to as being available at “http://eegstudy.org/prepcode”. This site no longer exists, and the code is no longer accessible via this link. The code has now been made available via an alternative link “https://github.com/VisLab/EEG-Clean-Tools”, and the article has been updated accordingly. A new **Data availability statement** has also been added to the article to highlight the way in which this data can be accessed as follows.

## Data Availability

Users can download the PREP pipeline as a freely available MATLAB library from https://github.com/VisLab/EEG-Clean-Tools. Users can cite all versions by using the DOI 10.5281/zenodo.22983431. The original version of this article has been updated.

